# Dynamic interplay between locus-specific DNA methylation and hydroxymethylation regulates distinct biological pathways in prostate carcinogenesis

**DOI:** 10.1186/s13148-016-0195-4

**Published:** 2016-03-15

**Authors:** Shivani N. Kamdar, Linh T. Ho, Ken J. Kron, Ruth Isserlin, Theodorus van der Kwast, Alexandre R. Zlotta, Neil E. Fleshner, Gary Bader, Bharati Bapat

**Affiliations:** Department of Laboratory Medicine and Pathobiology, University of Toronto, Toronto, ON Canada; Mount Sinai Hospital, Lunenfeld-Tanenbaum Research Institute, Toronto, ON Canada; The Donnelly Centre for Cellular and Biomolecular Research, University of Toronto, Toronto, ON Canada; Department of Pathology, University Health Network, University of Toronto, Toronto, ON Canada; Department of Surgery and Surgical Oncology, Division of Urology, University Health Network, University of Toronto, Toronto, ON Canada; Division of Urology, University Health Network, Toronto, ON Canada

**Keywords:** 5-Hydroxymethylcytosine, 5-Methylcytosine, Whole-genome, Prostate cancer, Cell lines, Integrative analysis

## Abstract

**Background:**

Despite the significant global loss of DNA hydroxymethylation marks in prostate cancer tissues, the locus-specific role of hydroxymethylation in prostate tumorigenesis is unknown. We characterized hydroxymethylation and methylation marks by performing whole-genome next-generation sequencing in representative normal and prostate cancer-derived cell lines in order to determine functional pathways and key genes regulated by these epigenomic modifications in cancer.

**Results:**

Our cell line model shows disruption of hydroxymethylation distribution in cancer, with global loss and highly specific gain in promoter and CpG island regions. Significantly, we observed locus-specific retention of hydroxymethylation marks in specific intronic and intergenic regions which may play a novel role in the regulation of gene expression in critical functional pathways, such as BARD1 signaling and steroid hormone receptor signaling in cancer. We confirm a modest correlation of hydroxymethylation with expression in intragenic regions in prostate cancer, while identifying an original role for intergenic hydroxymethylation in differentially expressed regulatory pathways in cancer. We also demonstrate a successful strategy for the identification and validation of key candidate genes from differentially regulated biological pathways in prostate cancer.

**Conclusions:**

Our results indicate a distinct function for aberrant hydroxymethylation within each genomic feature in cancer, suggesting a specific and complex role for the deregulation of hydroxymethylation in tumorigenesis, similar to methylation. Subsequently, our characterization of key cellular pathways exhibiting dynamic enrichment patterns for methylation and hydroxymethylation marks may allow us to identify differentially epigenetically modified target genes implicated in prostate cancer tumorigenesis.

**Electronic supplementary material:**

The online version of this article (doi:10.1186/s13148-016-0195-4) contains supplementary material, which is available to authorized users.

## Background

Tumorigenesis is regulated by a cascade of genetic and epigenetic alterations, with aberrant cytosine base methylation (5mC) acting as one of the key defining characteristics of tumor cells. CpG islands (CGIs), characterized by clusters composed of cytosine nucleotides followed by guanines, are usually unmethylated in normal conditions. However, in cancer, locus-specific hypermethylation of CGIs, particularly in the promoter regions of tumor suppressor genes, results in their loss of expression [1–3]. Simultaneously, genome-wide global hypomethylation of repetitive sequences in tumors results in genomic instability, promoting chromosomal rearrangement and the reactivation of transposable elements [4, 5].

5-Hydroxymethylated marks (5hmC) were first characterized in mammalian genomes as transient intermediates in the process of DNA demethylation [6]. However, the recent discovery of 5hmC as a stable epigenetic mark that also shows global loss in solid tumors and hematological malignancies [7, 8] has opened up new avenues for investigation into the dynamics of epigenetic regulation in cancer. 5hmC shows striking differences in distribution patterns among human tissues, exhibiting very high content in the brain and low content in the blood, spleen, and placental tissue [9, 10]. Genomic 5hmC distribution also differs by region, showing enrichment at exon-intron boundaries, exons, promoters, and enhancer elements [11–14]. Generally, the presence of 5hmC marks is associated with increased expression [14, 15]; however, the role of promoter hydroxymethylation in regulating expression may differ based on cell type [16].

Several pieces of evidence suggest a key role for 5hmC in governing tumorigenesis. Firstly, the genome-wide loss of 5hmC in cancer cannot be completely explained by the corresponding global loss of 5mC, indicating an independent role for 5hmC alterations in tumors [7]. Secondly, 5hmC correlates directly with differentiation state in cells during development, and its loss may thus dispose tumor cells toward uncontrolled proliferation [17, 18]. Furthermore, TET enzymes, which oxidize 5mC to produce 5hmC, often exhibit mutations or transcriptional downregulation in many different types of cancers, especially in hematological malignancies and gliomas [18–21]. The dioxygenase activity of TET proteins is dependent on the presence of α-ketoglutarate, which acts as a catalytic cosubstrate for 5hmC production [22]. Intriguingly, isocitrate dehydrogenase enzymes 1 (*IDH1*) and 2 (*IDH2*), which are normally able to produce α-ketoglutarate through the decarboxylation of isocitrate, are mutated in many human cancers. *IDH* mutations not only inhibit their ability to produce α-ketoglutarate but result in the production of the oncometabolite 2-hydroxyglutarate (2HG), which is able to directly inhibit the activity of *TET* proteins [20, 22, 23].

Thus, 5hmC patterning across the tumor genome may act as a hallmark of cancer development and progression. However, the locus- and gene-specific roles of 5hmC and their significance in tumorigenesis have not yet been well characterized in non-neuronal solid tumors, including prostate cancer (PCa).

PCa is the most common malignancy and the second highest cause of death from cancer in men worldwide [24]. Currently, the gold standard for PCa diagnosis is prostate-specific antigen (PSA) testing. However, due to its high false positive detection rate and the inability of PSA levels to differentiate between indolent and aggressive disease, the widespread usage of PSA screening has resulted in frequent overdiagnosis and overtreatment of the disease, an issue made critical by the significant morbidity associated with radical treatment [25–27]. This issue is further complicated by extensive tumor multifocality and heterogeneity. A majority of PCa patients present with multiple nonclonal foci of disease, several of which may possess differential histologic grades. Thus, genetic heterogeneity in PCa is not only widespread between patients but also within single prostate tumor specimens. Individual patients may thus possess multiple distinct genomic profiles at each tumor focus, complicating PCa diagnosis, prognostication, and development of treatment strategies [28–30].

Epigenetic alterations also contribute to PCa tumor heterogeneity, with locus-specific variability in cytosine base methylation (5mC) occurring concurrently with copy-number alterations in primary prostate tumors [31]. Although global hypomethylation changes are a late event in PCa, associated with metastatic progression [32], promoter hypermethylation is an early and frequent event in prostate carcinogenesis. Hypermethylation-induced silencing of the tumor suppressor glutathione S-transferase P1 gene (*GSTP1*) is the most frequent DNA aberration in PCa, while promoter hypermethylation of *GSTP1*, *RASSF1A* (Ras association domain family 1 isoform A), and *APC* (adenomatous polyposis coli) correlates with PCa stage [33, 34].

Thus, comprehensive characterization of the 5hmC modifications underlying PCa development and, consequently, the mechanisms of epigenetic regulation in PCa may help to address these issues. Investigation of differential patterns of 5hmC and 5mC distribution across genomic features (intergenic, intronic, exonic, promoter, DNAse I hypersensitive site, or CGI regions) could provide important insights into prostate cancer pathogenesis.

In this study, we aim to determine the biological role of aberrant 5hmC patterning in regulating biological pathways and key candidate genes in prostate tumorigenesis. We have used a whole-genome next-generation sequencing strategy to investigate the dynamic interplay between locus-specific 5mC and 5hmC marks and their relationship with altered gene expression, performing one of the first integrative analyses of its kind in prostate cancer. Our data shows that 5hmC marks display differential effects on gene regulation based on locus-specific changes unique to cancer cells and that it may play a critical role in governing central tumorigenic pathways related to signaling and cellular proliferation in prostate cancer.

## Results

### Genome-wide locus-specific hypermethylation and global reduction of hydroxymethylation occurs in prostate cancer cells compared to normal prostate

**Fig. 1:**
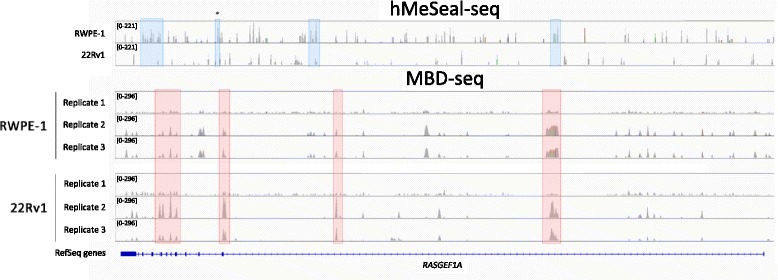
Locus-specific differential distribution of methylation and hydroxymethylation in normal prostate versus prostate cancer cell lines. Linear representation of hMeSeal-seq (*top*) and MBD-seq (*bottom*) peaks in representative normal prostate (RWPE-1) or prostate cancer (22Rv1) cells across the representative gene RasGEF domain family, member 1a (*RASGEF1A). Top:* generalized and locus-specific hypohydroxymethylation in 22Rv1. *Blue boxes* indicate representative regions exhibiting strong hydroxymethylation peaks in RWPE-1 and weak or absent hydroxymethylation peaks in 22Rv1. *Asterisk* indicates hydroxymethylated region validated by hMeSeal-qPCR testing. *Bottom:* locus-specific differential methylation between cell lines. *Red boxes* indicate representative regions showing strong methylation peaks in 22Rv1 and weaker peaks in RWPE-1. Note that certain regions also exhibit locus-specific methylation in RWPE-1 and not in 22Rv1

In order to investigate the distribution of 5mC and 5hmC in the prostate cancer genome, we performed next-generation sequencing (NGS) following methyl-binding protein capture (MBD-seq, *n* = 3) and hydroxymethyl-selective chemical labeling (hMeSeal-seq, *n* = 1) in a representative normal prostate tissue-derived cell line (RWPE-1) and a xenograft-derived prostate carcinoma cell line (22Rv1). Profiling of these epigenetic marks across RefSeq-annotated genes revealed dramatic differential hypermethylation across all genomic features in 22Rv1 as compared to RWPE-1, with gene expression lowering in concordance with increased methylation peak density. In contrast, marked global loss of absolute 5hmC was noted across the 22Rv1 genome (Additional file 1: Figure S1), while locus-specific alterations of both marks could be observed across genes (Fig. 1).

### 5mC and 5hmC show differential locus-specific abundance in prostate cancer

**Fig. 2:**
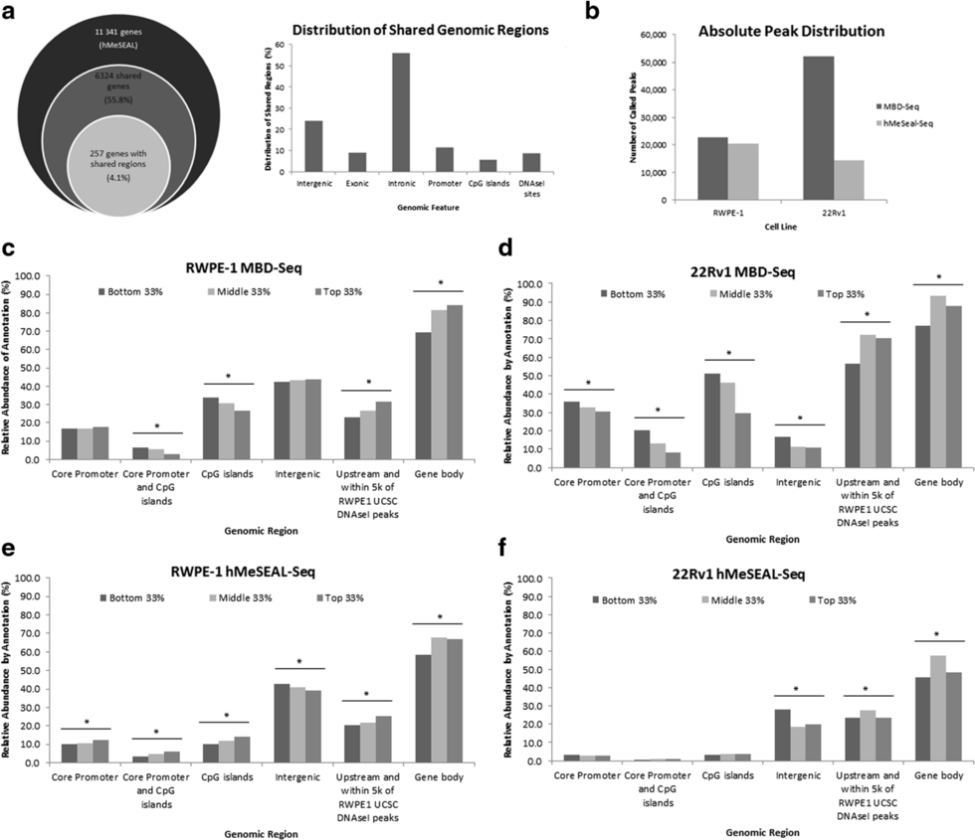
Correlation between locus-specific methylation or hydroxymethylation and gene expression. **a** Validation of hMeSeal-Seq results by hMeDIP-Seq. *Left*: 62.6 % of genes detected as hydroxymethylated in RWPE-1 by hMeSeal were also detected as hydroxymethylated using hMeDIP, while 2.3 % of peaks from these genes were called in exactly the same chromosomal location using both methods. *Right*: genomic feature distribution for peaks called in the same regions in both hMeDIP- and hMeSeal-seq. Some features may overlap each other, and thus one peak may be accounted for more than once. **b** Total peaks called from sequencing data in normal and prostate cancer cell lines. Peaks called across three replicates for MBD-Seq or within one single replicate for hMeSeal-seq. **c**–**f** Differentially methylated (**c**, **d**) and differentially hydroxymethylated (**e**, **f**) regions for both cell lines stratified by genomic feature. *Bar graphs* depict the relative abundance of each mark within each of three expression tiers from microarray analysis. *Asterisks* represent significant *p* values (*p* < 0.05, chi-square test for trend) indicating correlation between the presence of 5mC or 5hmC marks in a given region and gene expression within each cell line. Upward trend of bars, when significant, indicates a positive correlation of a locus-specific mark with gene expression. Significant downward trend indicates negative correlation

As locus-specific 5hmC is poorly characterized in prostate cancer, and due to the lowered distribution of 5hmC in cell culture [35], we first set out to determine the validity of our cell line hydroxymethylation profiling. hMeSeal-seq samples chosen for analysis displayed significant normalized read enrichment compared to input samples (Additional file 1: Figure S2), while dot blot analysis of genomic DNA from both cell lines showed corresponding evidence of 5hmC (Additional file 1: Figure S3). Furthermore, the proportion of promoter, intergenic, and CGI hydroxymethylation in both cell lines is significantly lower than one would expect from random genomic distribution of the 5hmC mark across CpGs, indicating the presence of locus-specific patterning in our samples (Additional file 1: Table S1). Validation of hMeSeal kit specificity was also performed via the testing of control oligomers (Additional file 1: Figure S4). Finally, in order to validate our 5hmC marks in RWPE-1, we performed hydroxymethylated DNA immunoprecipitation sequencing (hMeDIP-seq) on a separate biological replicate (*n* = 1) from the same normal cell line. Specificity of the hMeDIP antibody was verified via internal spike-in of control sequences (Additional file 1: Table S2). We found that 55.8 % of hydroxymethylated gene regions (6324 unique genes) detected by our hMeSeal technique were also detected in hMeDIP-seq, of which 4.1 % (representing 257 genes) bore 5hmC peaks with at least partial overlap in exactly the same 0.48–1.5-kbp regions (Fig. 2a). hMeDIP-seq samples were sonicated separately from hMeSeal-Seq samples, resulting in a different pattern of random shearing and making it highly unlikely that the observed overlap was generated by random chance. Peak distribution in both techniques did not significantly differ in all genomic features with the exception of DNAse I hypersensitive sites (DHSs), further confirming our hMeSeal-seq results (Additional file 1: Figure S5).

Next, we compared the abundance of differential methylation and hydroxymethylation peaks proximal to RefSeq genes between both cell lines (Fig. 2b), validating the specificity of MBD capture through quantitative PCR (qPCR) (Additional file 1: Figure S6). 22Rv1 had more than twice the number of differentially methylated annotated peaks or differentially methylated regions (DMRs) compared to RWPE-1. However, the locus-specific distribution of methylation marks differed between cell lines, with significant increases in the proportion of 5mC marks in CGIs and DHSs in cancer (Additional file 1: Figure S7A). DHSs are indicative of open chromatin and gene regulatory elements [36], and we obtained this dataset for RWPE-1 only. We observed significantly less proportional promoter methylation in 22Rv1 as compared to RWPE-1. These findings contrast with typical patterns of promoter hypermethylation in cancer and may potentially indicate site-specific derepression of certain genes. In contrast, the proportion of promoter CGI methylation was significantly greater in 22Rv1 than in RWPE-1, in accordance with the conventional methylation paradigm in cancer.

22Rv1 differential hydroxymethylation was significantly reduced in both overall peak number and proportionality of enrichment in exonic, promoter, CGI, and RWPE-1 DHS genomic features. However, proportional levels of intronic hydroxymethylation did not significantly differ between cell lines. Intriguingly, proportional intergenic hydroxymethylation was significantly greater in cancer compared to normal cells (Additional file 1: Figure S7B). Furthermore, pathway-based analysis of intergenic hydroxymethylation revealed extensive differential functional enrichment between normal and cancer cell lines, suggesting a putative role for intergenic 5hmC in the aberrant upregulation of proliferation androgen receptor signaling in cancer. While most literature to date has focused on pathological 5hmC loss, our results suggest a putative functional role for the presence or gain of hydroxymethylation marks in cancer.

### A novel negative correlation of 5hmC with expression in gene-proximal intergenic regions

In order to determine the effect of the observed differential epigenetic mark distribution in 22Rv1 on gene regulation, we performed integrative analysis correlating (hydroxy)methylation-enriched genes stratified by genomic feature to gene expression levels obtained from publicly available microarray data for RWPE-1 [Gene Expression Omnibus (GEO) Accession: GSM375783] (*n* = 1) and 22Rv1 [GEO Accession: GSE36135] (*n* = 3). We divided the expression data into three equal tiers representing low, median, and high relative expression and correlated genes significantly differentially enriched for either 5mC or 5hmC marks to expression within each tier (Additional file 1: Table S3 and S4).

We found gene expression to exhibit negative correlation with methylation enrichment in CGIs (both within and outside core promoter regions) and positive correlation with methylation in genic regions and regions within 5 kbp of UCSC RWPE-1 DHSs in both cell lines, as expected (Fig. 2c, d). Correlations between 5mC and expression tended to be more robust in the cancer cell line, with strong negative correlation to expression also observed for intergenic and promoter methylation alone in 22Rv1.

Significantly, we observed several novel correlations between hydroxymethylation and gene expression in the prostate. Hydroxymethylation enrichment in promoters, CGIs, and CGIs within the core promoter region correlated positively with gene expression in normal prostate, while a similar correlation with expression in these features was not observed in cancer (Fig. 2e–f). These findings contrast with the proportional gain of promoter and CGI 5hmC observed in pancreatic cancer [37]. Intriguingly, however, we observed a novel negative correlation between hydroxymethylation enrichment in intergenic regions and expression of the genes with transcription start sites (TSS) closest to the 5hmC mark. This correlation was strengthened in cancer, in contrast to the weakening of all other 5hmC expression trends. Furthermore, while 5hmC in genic regions and within 5 kbp of UCSC RWPE-1 DHSs exhibited linear positive correlation with gene expression in normal prostate (similar to 5mC), this correlation was strongly altered in prostate cancer, where the presence of hydroxymethylation in these features showed nonlinear correlation with expression in the median tier. This trend persisted despite the significant decrease of 5hmC in cancer observed in exonic and DHS-proximal regions and may indicate altered functionality of 5hmC marks in prostate cancer.

The same correlation was also performed for RWPE-1 hMeDIP-seq data (Additional file 1: Table S5), where intergenic, genic, and DHS-proximal trends from hMeSeal-seq were strongly corroborated (Additional file 1: Figure S8).

### Absence, gain, and retention of 5hmC differs significantly by genomic region between normal and cancer cell lines

We examined absolute peak regions where 5hmC marks detected in RWPE-1 either overlapped with peak regions detected as having 5mC marks in 22Rv1 (5hmC “absence”) or 5hmC marks in 22Rv1 (5hmC “retention”), as well as peaks where 5mC marks in RWPE-1 overlapped with 5hmC marks in 22Rv1 (5hmC “gain”). We then tested these peak regions, stratified by genomic location, to determine genomic features where the frequency of occurrence of these overlapping peaks could not be explained by either random change in RWPE-1 epigenetic marks or by random distribution of 22Rv1 marks within each genomic feature (Additional file 1: Table S6).

Peaks located within exonic, promoter, or RWPE-1 DHS genomic features exhibited greater 5hmC depletion than expected from the proportion of global RWPE-1 5hmC or 22Rv1 5mC; however, they did not significantly differ from expected values for random 5hmC retention or gain in cancer (Table 1). In other words, peaks within these features were far more likely to exhibit absence of 5hmC in cancer than to gain or retain it (Additional file 1: Table S7), suggesting that loss of 5hmC within these specific features may be especially important in tumorigenesis. In contrast, intergenic regions in cancer showed far less gain of 5hmC than expected, despite being the only region in which proportional 5hmC absence in 22Rv1 was non-significant. Intriguingly, both absence and gain of 5hmC was greater than expected within CGIs, while intronic regions showed significantly less gain of 5hmC than expected. Unlike all other features tested, however, the proportions of peak regions showing 5hmC absence, gain, or retention in intronic regions did not significantly differ from each other. These findings further indicate that changes in 5hmC regulation and functionality in cancer differ between specific genomic features. However, despite the significant changes in 5hmC absence in both intronic and exonic regions, genic changes (comprised of the sum of intronic, exonic, and UTR regions) in 5hmC status were not found to be significantly different from random distribution in any category.

**Table 1:** Locus-specific hydroxymethylation changes in cancer. 5hmC changes were described by the overlap of 5mC and/or 5hmC marks between cell lines. Overlapping 5mC or 5hmC marks were compared to the overall distribution of each mark within each cell line to determine significant differences

Differential 5mC/5hmC status in PCa	Prostate cell type (normal vs. cancer)	Overlapping methylation marks	Genomic region						
			Intergenic	Genic	Exonic	Intronic	Promoter	CpG Island	RWPE-1 DNaseI hypersensitive sites
5hmC “Absent”	RWPE-1	5hmC	0.051	0.666	<0.0001^*,**^	0.005^*,***^	<0.0001^*,**^	<0.0001^*,**^	<0.0001^*,**^
	22Rv1	5mC	<0.0001^*^	0.311	0.002^*,**^	0.001^*,***^	<0.0001^*,**^	<0.0001^*,**^	<0.0001^*,**^
5hmC “Retained”	RWPE-1	5hmC	0.302	0.01	0.039	0.169	0.487	0.593	0.067
	22Rv1	5hmC	0.013	0.547	0.452	0.834	0.115	0.001^*^	0.944
5hmC “Gained”	RWPE-1	5mC	0.003^*,***^	0.975	0.175	0.834	0.5	<0.0001^*,**^	0.007^*^
	22Rv1	5hmC	<0.0001^*,***^	0.026	0.025	0.538	0.71	<0.0001^*,**^	0.511

*Significant *p* values as determined by the chi-square test with Bonferroni correction for multiple testing applied; **5hmC is significantly more likely to be absent, gained, or retained within the given region than expected; ***5hmC is significantly less likely to be absent, gained, or retained within the given region than expected

### Biological pathways regulated by 5mC and 5hmC marks differ significantly between normal prostate and prostate cancer

**Fig. 3:**
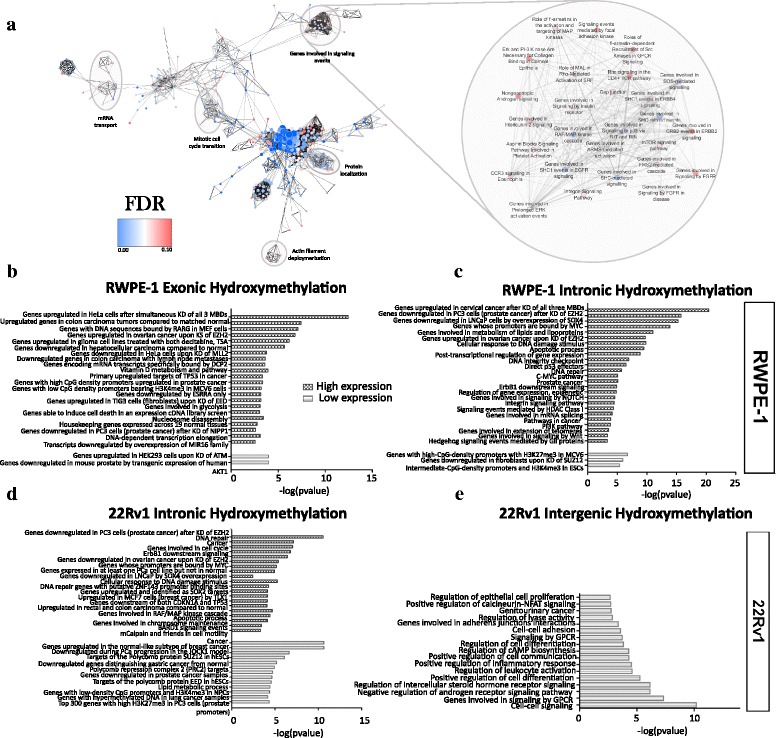
Pathway regulation by methylation and hydroxymethylation marks in normal prostate and prostate cancer. **a** Representative example of an enrichment map generated using Cytoscape depicting annotations significantly enriched for genes exhibiting intronic hydroxymethylation and highest tier expression levels in 22Rv1 alone as compared to all genes detected in both cell lines via hMeSeal-Seq. Annotated using the GREAT hypergeometric test over regions (*p* value < 0.05, FDR < 0.1, Jaccard’s similarity coefficient < 0.25). *Left*: select clusters of related pathways (nodes) in the enrichment map are *highlighted* (clusters identified using MCODE). *Right:* zoom in to signaling pathway enrichment map cluster. *Below:* pathway enrichment annotations from GREAT for hydroxymethylation within RWPE-1 cells, overlapping (**b**) exonic or (**c**) intronic regions and hydroxymethylation within 22Rv1 overlapping (**d**) intronic or (**e**) intergenic regions*.* Pathways further represented by genes within the highest (high expression) and lowest tier of expression (low expression)

We performed pathway analysis on genes enriched in either 5mC or 5hmC differential marks within each tier of expression and genomic feature. To identify differential gene regulation by each mark in normal versus cancer, we compared the most significant and specific annotation terms (defined by grouping similar enriched pathways using the MCODE clustering algorithm and manually labeling the groups), identified via the Genomic Regions Enrichment of Annotation Tool (GREAT), between cell lines (hypergeometric test over regions; false discovery rate (FDR) < 0.05). Individual enrichment maps were generated for each feature and expression tier within each cell line prior to pathway comparison (Fig. 3a).

Pathway enrichment for differentially methylated genes was primarily observed within CGIs for 22Rv1, where multiple genes exhibiting CGI methylation and functionally enriched for annotations related to the regulation of cell proliferation, adenylate cyclase activity, and lyase activity displayed lowered expression in cancer compared to normal (Additional file 1: Figure S9A). In contrast, annotations for differentially methylated genes in exonic regions were primarily enriched for core cellular functions in both cell lines (Additional file 1: Figure S9B–S9C). Similarly, although differential intronic methylation patterns showed enrichment for core pathways in both cell lines (Additional file 1: Figure S10A–S10B), most genes did not exhibit concordant differential expression tiers from the normal to the cancer cell line.

Contrary to our expectations, 5hmC expression pathway trends paralleled 5mC expression trends within CGI and exonic regions in cancer. While hydroxymethylated genes in CGIs remained unenriched for relevant pathways in RWPE-1 cells (Additional file 1: Figure S11A), annotations related to Akt, Wnt, and mTor signaling, as well as inhibitory p53 feedback loops, were abundant within highly expressed genes exhibiting CGI hydroxymethylation in 22Rv1 (Additional file 1: Figure S11B). However, all genes involved were also highly expressed in normal cells.

Exonic hydroxymethylation terms, in contrast, were not significantly enriched in 22Rv1 (data not shown) but displayed enrichment within the highest tier of expression for a diverse range of pathways (including housekeeping genes expressed across 19 normal tissues, genes able to induce cell death, and vitamin D metabolism) within RWPE-1 (Fig. 3b). Furthermore, many of these genes showed reduced expression in our cancer cell line, which may indicate a role for exonic 5hmC in the normal regulation of core cellular functions.

Similar to intronic 5mC-expression profiling patterns, hydroxymethylated intronic regions in RWPE-1 showed few significant pathway annotations within the lowest tier of expression (Fig. 3c), unlike 22Rv1 (Fig. 3d). However, extensive enrichment was observed within the highest expression tier for pathways related to DNA damage repair, integrity checkpoints, apoptosis, and direct p53 effectors (Fig. 3c). Interestingly, annotations related to prostate cancer, the c-Myc pathway, and telomere extension were also observed within the normal cell line within the highest expression tier. As the RWPE-1 cell line has been immortalized through the use of human papillomavirus, these pathways may be indicative of aberrations incurred from the immortalization process. In cancer, significant pathway enrichment was seen in both low and high expression tiers, with polycomb repressive complex 2 (PRC2) targets and genes known to be downregulated in PCa enriched in the lowest expression tier while pathways related to cell motility, BARD1 and ErbB1 signaling, and genes known to be enriched in PCa specifically were observed in the highest expression tier (Fig. 3d). Interestingly, DNA repair and apoptotic pathway enrichment was also seen within this tier in cancer, although most genes involved were expressed at the same level in both normal and PCa cells.

Perhaps most intriguingly, intergenic regions exhibited pathway enrichment within the lowest tier of expression unique to 22Rv1 cells. Strikingly, many of these pathways were associated with tumorigenic functions (Fig. 3e), suggesting a putative role for intergenic hydroxymethylation in the downregulation of inflammation and cellular adhesion and the upregulation of epithelial cell proliferation. Of particular interest, however, was the presence of 5hmC enrichment in pathways governing the negative regulation of androgen signaling. Furthermore, differentially hydroxymethylated genes within this pathway, including the PCa-inhibitory genes secreted fizzled-related protein 1 (*SFRP1)* and Dab, mitogen-responsive phosphoprotein homolog 2 (*DAB2*), exhibited significantly lower expression in cancer than in the normal cell line. In sharp contrast, RWPE-1 showed few significant functional annotations for low-tier intergenic hydroxymethylation marks (Additional file 1: Figure S12). Overall, exonic and intergenic regions showed the most striking differential pathway enrichment between normal and cancer cells for both 5mC and 5hmC marks. Differentially hydroxymethylated genes from high-level exonic pathways in RWPE-1 and low-tier intergenic pathways in 22Rv1 exhibited large differences in expression between cell lines, potentially indicating a critical role for aberrations in exonic and intergenic patterning of 5mC and 5hmC marks in cancer.

### Locus-specific 5mC and 5hmC marks share a novel cooperative role in the regulation of functional pathways

**Fig. 4:**
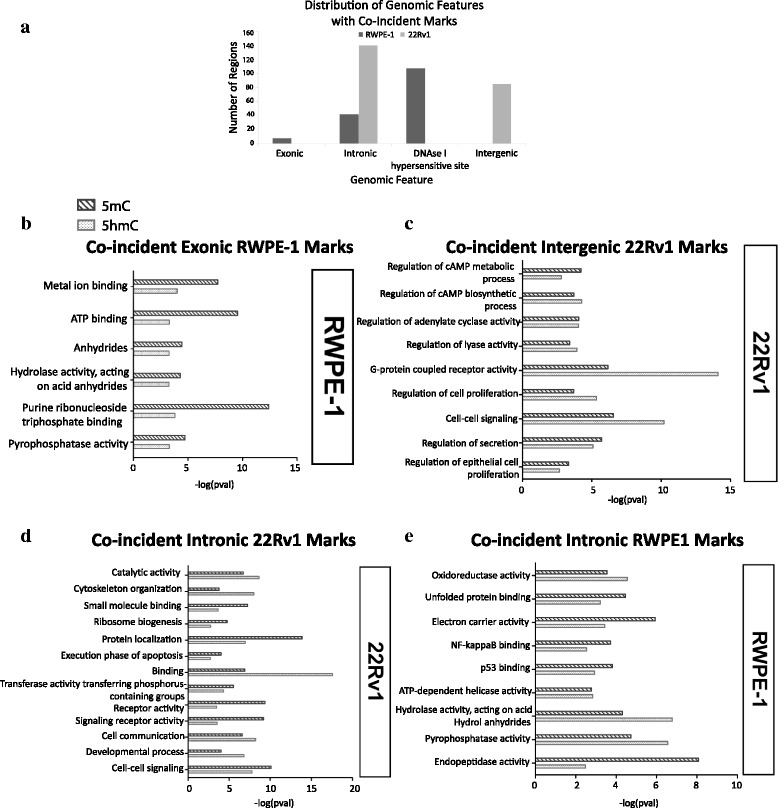
Locus-specific co-enrichment of methylation and hydroxymethylation marks in normal prostate versus prostate cancer. **a** Genomic distribution of 5hmC marks exhibiting co-incidence with 5mC in normal prostate versus prostate cancer cells. Co-incident marks were defined as precise regions within the same cell line containing both 5mC and 5hmC marks which exhibited at least partial overlap. **b**–**e** Pathway enrichment annotations from GREAT for co-incident marks in (**b**) RWPE-1 exonic regions, (**c**) 22Rv1 intergenic regions, or intronic regions in (**d**) 22Rv1 or (**e**) RWPE-1 cells. Log *p* values for methylation marks (*top*) and hydroxymethylation marks (*bottom*) are shown

We compared biological pathway annotations for genes enriched in both 5mC and 5hmC within each cell line by expression tier and genomic feature in order to determine whether or not intercellular differences observed in the type of epigenetic modifications resulted in significant differences in pathway regulation (Additional file 1). Surprisingly, we found that a significantly higher proportion of pathways enriched for both marks in either cell line showed intracellular co-enrichment of 5hmC and 5mC within the same expression tier on the same gene (*p* < 0.0001). While promoter and CGI methylation and hydroxymethylation marks governed completely different pathways, genes with intronic 5hmC marks exhibited strong 5mC and 5hmC co-enrichment for specific cellular functions in both cell lines (Fig. 4a). Furthermore, while gene-specific methylation and exonic hydroxymethylation were observed to co-regulate pathways related to basic cellular functionalities such as ATP binding and hydrolase activity in RWPE-1 (Fig. 4b), this co-regulation was entirely lost in 22Rv1, mirroring the loss of exonic 5hmC marks in cancer. Intriguingly, co-occurrence of 5mC with intergenic 5hmC epigenetic marks was observed in 22Rv1, but not in RWPE-1, coinciding with high-level functional annotations in the lowest tier of expression including signaling, regulation of cellular proliferation and cAMP biosynthesis regulation in cancer (Fig. 4c).

Additionally, we found that most hydroxymethylated intronic pathways showing concomitant 5mC enrichment in cancer were related to basic cellular functions, such as binding, protein localization, and cytoskeletal organization, within each expression tier (Fig. 4d). This observation contrasts sharply with hydroxymethylated intronic co-enrichment pathways in RWPE-1, where basic processes predominated in the middle and lowest expression tiers (Fig. 4e). However, annotations for genes exhibiting both marks within the highest tier of expression were enriched for multiple highly specific processes, including pathways related to the electron transport chain as well as binding of both NF-κB and p53. This data provides novel evidence for the highly locus- and gene-specific dysregulation of 5hmC in governing oncogenesis.

### Genes involved in key differential biological processes are detectable via hMeSeal-qPCR

**Fig. 5:**
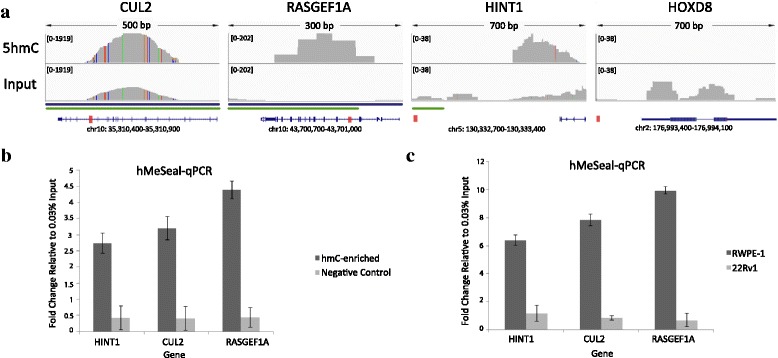
Validation of differentially hydroxymethylated regions (DHMRs) via hMeSeal-qPCR. **a** *Top*: IGV Genome Browser representation of absolute hydroxymethylation peaks called in single replicate from hMeSeal-seq data within regions showing hydroxymethylation in both hMeSeal- and hMeDIP-seq and compared to unenriched input control for three representative genes for validation: *CUL2*, *RASGEF1a*, and *HINT1*, and *HOXD8* negative control. Peak strength indicated by height of representative peak within replicate or input control (scale shown in *square brackets*). *Colored vertical bars* within peaks represent differences between sequenced bases and the hg19 reference genome. *Bottom*: *Blue horizontal bars* indicate the presence of introns. *Green horizontal bars* indicate CGIs. *Red boxes* indicate the position of the hMeSeal-qPCR amplified segment depicted relative to the overall location of the gene (figure not to scale). Gene diagram adapted from UCSC Genome Browser. **b** Validation of hydroxymethylation gene enrichment for RWPE-1. Enrichment of hydroxymethylation detected via hMeSeal-qPCR for candidate genes, *CUL2*, *RASGEF1a*, and *HINT1*, within RWPE-1 compared to *HOXD8* negative control and normalized relative to 0.03 % input control using the ΔΔC_t_ method (mean values ± standard deviation, *n* = 3). All genes were compared to negative hMeSeal control reaction performed without UDP-azide-glucose and representing nonspecific binding. **c** Hydroxymethylated gene enrichment for RWPE-1 versus 22Rv1. Differential detection of genes identified as uniquely hydroxymethylated in RWPE-1, but not 22Rv1, by hMeSeal-seq via hMeSeal-qPCR compared to negative control hMeSeal reaction and normalized relative to 0.03 % input control using the ΔΔC_t_ method (mean values ± standard deviation, *n* = 3)

We chose representative genes for validation which were localized to hydroxymethylated regions detected by both hMeSeal-seq and hMeDIP-seq in RWPE-1, were not hydroxymethylated in 22Rv1, and overlapped RWPE-1 DHSs (Additional file 1: Table S8). Selected genes were not methylated in either cell line (Additional file 1: Figure S13). Cullin 2 (*CUL2*) bore methylation marks overlapping hydroxymethylation peaks in RWPE-1, but not in 22Rv1; in contrast, RasGEF domain family member 1a (*RASGEF1a*) and histidine triad nucleotide-binding protein 1 (*HINT1*) were methylated in the same region in 22Rv1 (exhibiting 5hmC “loss” in cancer while not being methylated in RWPE-1). *RASGEF1a* and *CUL2* were located in intronic regions, while *HINT1* refers to an intergenic 5hmC peak located most closely to the TSS of *HINT1*. Lastly, all genes chosen possessed robust and clear 5hmC enrichment peaks within the overlapping regions, with corresponding absent or low peaks in input samples (Fig. 5a).

In order to validate the specificity of our sequencing data and quantify the relative 5hmC levels, we performed qPCR with standard curve protocol following hMeSeal enrichment of genomic RWPE-1 and 22Rv1 DNA. We used primers flanking the identified enrichment peaks for *CUL2*, *RASGEF1a*, and *HINT1*, as well as the negative control gene homeobox D8 (*HOXD8*). *HOXD8* was found to be strongly methylated in RWPE-1 (Additional file 1: Figure S14) and was not hydroxymethylated in either cell line. Negative control capture reactions lacking UDP-azide-glucose followed by qPCR were performed simultaneously across three technical replicates. Enrichment of *HOXD8* was not detected via qPCR, confirming the specificity of our capture reaction. *CUL2*, *RASGEF1a*, and *HINT1* positive and negative control qPCR reactions were normalized to *HOXD8* negative control using the ΔΔC_t_ method, obtaining fold enrichment values of 2.73×, 3.20×, and 4.39×, respectively (Fig. 5b), while enrichment was not detectable in 22Rv1 (Fig. 5c). These findings validate our hMeSeal-seq results and show that locus-specific detection of 5hmC enrichment is possible in the prostate and prostate cancer cell lines.

## Discussion

Recent studies have provided direct evidence supporting a key role for 5hmC in regulating transcription, both through the structural influence of locus-specific 5hmC hemimodification on transcription factor binding [38] and the active recruitment of 5hmC “readers” regulating transcription as described in murine retinal development [39]. In hematological cancers such as acute myeloid leukemia, locus-specific loss of 5hmC has been negatively correlated with gene expression in intronic, genic, promoter, and distal regulatory regions [40]. However, previous studies in solid tumors have focused primarily on mapping genome-wide loss of hydroxymethylation independently of genomic features [7, 9, 21]. Exceptions include pancreatic cancer, in which a positive association was found between promoter or gene body 5hmC enrichment and gene expression in both normal and cancer cells [30], and in colon cancer, in which promoter hydroxymethylation in normal cell lines was found to render them resistant to oncogenic methylation gain [13], and also melanoma, in which enrichment of oncogenic functions was found in genes losing 5hmC marks and gaining 5mC marks in cancer [41].

In prostate cancer, 5hmC has been found to be both globally depleted and inversely correlated with cell proliferation [7, 37] and has recently been implicated as an inhibitory mark downregulating gene expression in metastatic PCa cell line models [42], in direct contrast to our findings. As the distribution of hydroxymethylation marks differs significantly between tissue types [9], this discrepancy may be reflective of differences between PCa models or may even be indicative of differential genomic feature-specific effects of 5hmC modification. To date, the locus-specific role of 5hmC modification in governing gene expression and functionality has not been identified in PCa. Furthermore, while oncogenic pathway enrichment has been found in regions exhibiting 5hmC gain in pancreatic cancer [37], our data did not reveal significant pathway enrichment for similar genes in our cell lines. This may be due to significant global loss of 5hmC marks in cell culture further impairing detection of hydroxymethylated regions in our PCa cell line [35] or an example of a tissue-specific 5hmC profile.

One important caveat of our cell line model is the rapid global loss of 5hmC observed by Nestor et al. in cell lines almost immediately upon culture and passaging [35]. High-passage mouse embryonic fibroblast cell lines and primary T cell cultures were found to lose 5hmC to such a degree that clear hydroxymethylation patterning was lost from the tissues or primary cells of origin. Loss of 5mC in culture, however, was much milder and allowed for the retention of patterning. Although global levels of 5hmC in our cell lines were low, it should be noted that our validation of hMeSeal-seq data via both genome-wide hMeDIP-seq and locus-specific hMeSeal-qPCR contraindicates the loss of clear 5hmC patterning within our cell line models.

The overall distribution of 5mC and 5hmC marks within each cell line showed an absolute methylation peak increase and hydroxymethylation peak decrease for all genomic features in cancer. However, the lack of significant intronic 5hmC difference between cell lines, coupled with our observation that intronic regions are the most likely to gain 5hmC in PCa, may indicate that intronic hydroxymethylation is both tightly regulated and potentially critical for basic cellular function. Similarly, the proportional gain of intergenic 5hmC and its status as the most likely feature to retain 5hmC in cancer may indicate either a lack of importance for intergenic 5hmC marks or, conversely, a key regulatory function in oncogenic transformation. However, the strong negative correlation between intergenic hydroxymethylation and gene expression in both cell lines, in addition to the significant pathway enrichment in genes bearing intergenic 5hmC and in the lowest tier of expression for terms related to androgen receptor regulation, inflammation, and cellular adhesion, provides strong support for the latter hypothesis. Our findings may indicate novel biological roles for intergenic 5hmC modifications located outside of regulatory regions and intron-specific 5hmC marks gained in cancer. Since many different factors can contribute to results from pathway-based analysis, further functional validation of implicated candidate genes will be performed using in vitro cell lines and animal models in order to verify these findings.

In contrast, although CGIs were found to be generally depleted for 5hmC marks in both of our cell lines, to such an extent that expression patterning was entirely lost in cancer, the extent of 5hmC depletion and gain within these features suggests that specific changes in CGI hydroxymethylation may be especially important for not only downregulation of genes in PCa but their upregulation as well. Interestingly, despite the significant exonic absence and intronic retention of 5hmC in cancer, genic regions exhibit no significant differences in 5hmC from random changes in either cell line, suggesting that the level of 5hmC retention in introns matches the magnitude of 5hmC depletion in exonic regions. Therefore, although many studies examine genic 5hmC alterations at a global level in development and disease, the observed distinct function of 5hmC within exons and introns in our samples underscores the need for further functional studies of 5hmC to be performed in a genomic feature-specific manner.

We found that functional mark distribution clearly suggested a distinct biological role for 5mC and 5hmC within our cell lines. Our analysis revealed several major biological pathways exhibiting differential epigenetic regulation in normal and cancer cell lines. Within 22Rv1 CGIs, 5hmC was positively correlated with genes involved in signaling processes promoting cell proliferation and migration and downregulating apoptotic p53 signaling, while 5mC was simultaneously negatively correlated with expression of genes regulating cyclic AMP (cAMP) generation. These observations reflect the contrasting relationships between CGI methylation or hydroxymethylation and expression.

Additionally, we observed distinct biological roles for 5mC and 5hmC marks between cell lines. Intronic hydroxymethylation was not significantly lost in our cancer cell line compared to normal, indicating that 5hmC may undergo specific redistribution in tumorigenesis. For example, while RWPE-1 intronic hydroxymethylation appears to positively regulate genes related to calpain I (*uCalpain*) activity in cell adhesion, 22Rv1 intronic 5hmC is enriched for pathways related to calpain II (*mCalpain*) involvement in cell motility. Intriguingly, almost all RWPE-1 hydroxymethylated genes involved in the tumor necrosis factor pathway exhibit lowered expression in 22Rv1, indicating a critical role for intronic 5hmC in governing this pathway. Due to the potentially confounding effects of immortalization in our cell lines, future functional analysis will be performed in tissue samples and in vivo models in order to further investigate the differential functionality implied by pathway analysis.

Many of the annotations observed for genes co-incident for 5hmC and 5mC marks in either cell line are related to basic cellular functions, which are often broad categories involving many genes [41, 43]. However, we also observed striking differences in both mark distribution and annotations between cell lines and genomic features with specialized pathways that are more likely to be indicative of true functional differences in epigenetic regulation.

Co-incidence of 5mC with exonic 5hmC was lost in 22Rv1 compared to normal, along with overall exonic 5hmC levels, representing a loss of co-incident marks in basic cellular pathways observed in RWPE-1. Intriguingly, this observation stands almost in direct contrast to intronic 5hmC and 5mC mark profiling in cancer, where intronic retention of both marks was seen for terms related to basic cellular functionality. This may possibly indicate their more essential role in basic cell survival than exonic modifications. Alternatively, it may also suggest that the genes involved in the cellular processes enriched for 5mC co-incidence with exonic 5hmC are more likely to exert functions inhibitory to tumor growth than those in intronic regions. Considering that overall 5hmC levels within introns are still lost in 22Rv1, an examination of noncoding RNA expression from intronic regions exhibiting differential epigenetic regulation in cancer might reveal the function of specific mark retention or gain in PCa.

The proportional increase of intergenic 5hmC in cancer, coupled with its robust correlation to low gene expression, may indicate a novel cooperative role for locus-specific 5hmC and 5mC in the regulation of tumor-suppressive functions in both normal and aberrantly modified PCa cell lines. Furthermore, this data suggests not only the precise dysregulation of nonessential 5hmC in intronic pathways related to tumor-suppressive functions in cancer but also that locus-specific intergenic gain of 5hmC may be used by tumor cells in conjunction with aberrant methylation elsewhere on the gene to actively suppress antitumor function.

In accordance with our novel findings regarding the role of intronic and intergenic hydroxymethylation in PCa, we chose two intronic genes—*RASGEF1a* and *CUL2*—and one intergenic gene—*HINT1*—for validation studies based primarily on the robustness of concordance between hMeDIP and hMeSEAL peaks, as well as lack of methylation and functionality in cancer.

Overexpression of *RASGEF1a*, an activator of the RAS family of oncogenes promoting cellular migration, is linked to oncogenesis in cholangiocarcinoma [44, 45]. The hydroxymethylation peak detected for *RASGEF1a* was located at the exon-intron boundary, a region known to exhibit neuronal 5hmC enrichment in the literature [11]. In normal cells, *HINT1* exerts tumor-suppressive effects through inhibition of the Wnt pathway and the promotion of both apoptosis and p53 expression and is known to undergo transcriptional silencing via promoter hypermethylation in colon and non-small cell lung cancer [46, 47]. Despite exhibiting the weakest peak of all three genes chosen for validation, the *HINT1-*proximal 5hmC peak was still robustly detected by hMeSeal-qPCR, confirming that intergenic 5hmC marks can also be reliably detected using our whole-genome sequencing strategy. Finally, *CUL2* was chosen as a representative validation gene due to the strength of its intronic hMeSeal-seq peak and its function as a component of the tumor-suppressive Von Hippel-Lindau complex, inhibiting uncontrolled angiogenesis via ubiquitination and degradation of hypoxia-inducible factor 1 alpha (*HIF1α*) [48]. As all three genes were able to be detected specifically in the normal cell line, we have verified the ability of our hMeSeal-Seq technique to reliably and specifically detect differentially distributed and weakly hydroxymethylated genes.

Since our strategy for selecting representative candidates was successful, we propose that further 5hmC candidates be selected in this manner. Key candidate genes involved in the regulation of critical oncogenic processes will be identified from region-specific central biological pathways exhibiting differential 5hmC modification based on hMeSeal-seq peak strength, function, annotation significance and relevance, putative association with TET enzymes, and differential gene expression between normal and cancer cell lines. Ultimately, these genes will be validated in primary tissue samples from both normal and early-stage cancer, as we expect that specific changes in 5hmC marks may be an early event in tumorigenesis.

## Conclusions

We demonstrate the highly locus-specific correlation of hydroxymethylation with gene expression in normal prostate cells, with a loss of robustness for correlation with positive expression in intergenic regions as well as a novel observation of increased robustness in negative expression correlation. Our findings indicate that loss of 5hmC in exonic, intronic, and CGI genomic regions, coupled with novel proportional hydroxymethylation gain in intergenic regions, may constitute important mechanisms of transcriptional repression in cancer development. Simultaneously, integrative pathway analysis correlating 5hmC to 5mC mark distribution, stratified by genomic feature, reveals their cooperative role in governing key biological pathways implicated in 5hmC-directed regulation of tumorigenesis. The selection of candidate genes exhibiting differential expression and epigenetic regulation from these functional pathways may provide novel avenues for the investigation of new and pre-existing biomarkers of cancer development.

Our locus-specific integrative analysis of hydroxymethylation marks is the first study of its kind to be performed in prostate cancer. Ultimately, our characterization of key cellular pathways exhibiting dynamic enrichment alterations for 5hmC or 5mC marks may not only allow us to identify novel biomarkers of disease but may also lead to the discovery of potential therapeutic target genes in PCa.

## Methods

### Cell culture and DNA extraction

Normal human prostate epithelial cell line, RWPE-1, was obtained from the American Type Culture Collection (ATCC). Human prostate cancer cell line, 22Rv1, was provided by Dr. E. Diamandis (Mount Sinai Hospital). RWPE-1 cells were cultured with Keratinocyte serum-free medium (K-SFM) (Invitrogen) supplemented with 0.05 mg/ml bovine pituitary extract (BPE) and 5 ng/ml human recombinant epidermal growth factor (EGF). 22Rv1 cells were cultured with RPMI 1640 (Mount Sinai Hospital) with 10 % fetal bovine serum (FBS). All cells were cultured as a monolayer and maintained in a humidified incubator at 37 °C with 5 % CO_2_. Genomic DNA was extracted from cells after trypsinization, using the QIAamp DNA Mini Kit (Qiagen) following the protocol provided.

### DNA methylation enrichment and next-generation sequencing: MBD-seq

Genomic DNA extracted from RWPE-1 and 22Rv1 cells was sonicated into approximately 100–300 bp fragments using a Vibracell Disrupter (SONICS). Sheared genomic DNA (4 μg) was incubated with methyl-CpG binding domain 2 (MBD2) protein and magnetic streptavidin beads provided by the MethylMiner Kit (Invitrogen). Bound DNA fragments were eluted with the highest concentration of NaCl buffer (2000 mM). Bound, unbound, and input DNA fragments were precipitated using MinElute PCR Purification Kit (Qiagen) with the final elution in UltraPure Distilled Water (Invitrogen). Bound and input DNA was quantified using Qubit 2.0 Fluorometer (Invitrogen). Bound, MBD2-enriched DNA (20 ng), and input samples (non-enriched) from 22Rv1 and RWPE-1 cells were submitted in triplicate biological replicates for library preparation (NEBNext® ChIP-Seq Library Prep Reagent Set for Illumina) and high-throughput sequencing using the HiSeq 2000 and 2500 (Illumina) at The Centre for Applied Genomics (The Hospital for Sick Children). Each library generated approximately 50 million paired-end reads with 5× coverage of CpGs.

### DNA hydroxymethylation enrichment by glucosylation and next-generation sequencing: hMeSeal-seq

Genomic DNA extracted from RWPE-1 and 22Rv1 cells was sonicated into approximately 100–300 bp fragments using a Q125 sonicator (QSonica). Sheared genomic DNA (10 μg) was specifically glucosylated at 5hmC by T4-phage β-glucosyltransferase and incubated with biotin and streptavidin beads provided by the Hydroxymethyl Collector Kit (Active Motif). The final elution of bound, unbound, and input DNA fragments were precipitated according to the protocol provided with the final elution in UltraPure Distilled Water (Invitrogen). Bound and input DNA was quantified using Qubit 2.0 Fluorometer (Invitrogen). Each replicate of 5hmC-enriched sample was prepared by pooling up to four hMeSeal reactions. Following, bound/glucosylated 5hmC-enriched DNA (15 ng) and input samples (non-enriched) from RWPE-1 and 22Rv1 cells were submitted in triplicates for library preparation (NEBNext® ChIP-Seq Library Prep Reagent Set for Illumina) and high-throughput sequencing using the HiSeq 2500 (Illumina) at The Centre for Applied Genomics (The Hospital for Sick Children). Each library generated approximately 50 million paired-end reads, with 10× coverage of CpGs.

### DNA hydroxymethylation enrichment by immunoprecipitation and next-generation sequencing: hMeDIP-seq

RWPE-1 genomic DNA from a separate biological replicate was submitted to Arraystar Inc. (Rockville, Maryland) where hMeDIP enrichment and NGS processing were performed. Genomic DNA was sonicated into approximately 200–600 bp fragments with a Bioruptor sonicator (Diagenode). Sheared genomic DNA (800 ng) was end-repaired, A-tailed, and ligated to single-end adapters following the standard Illumina genomic DNA protocol. Agarose size-selection was used to remove unligated adapters. Adaptor-ligated DNA was heat-denatured at 94 °C for 10 min and rapidly cooled on ice. Following, the denatured DNA was immunoprecipitated with 1 μL mouse monoclonal anti-5-hmC antibody (Diagenode) overnight at 4 °C, with rocking agitation in 400 μL immunoprecipitation (IP) buffer (0.5 % BSA in PBS). Five immunoprecipitation washes were performed with ice-cold IP buffer. A nonspecific mouse IgG immunoprecipitation was performed as a negative control. Washed beads were resuspended in TE buffer (with 0.25 % SDS and 0.25 mg/mL proteinase K) for 2 h at 65 °C and then cooled to room temperature. Bound/immunoprecipitated and unbound DNA fragments were purified using MinElute columns (Qiagen) and eluted in 16 μL of elution buffer (Qiagen). Immunoprecipitated fragments (5 μL) were subjected to 14 cycles of PCR using single-end PCR primers (Illumina). The resulting reactions were purified with MinElute columns (Qiagen), after which a final size selection (300–700 bp) was performed by electrophoresis in 2 % agarose. Libraries were quality controlled by Agilent 2100 Bioanalyzer. An aliquot of each library was diluted in elution buffer (Qiagen) to 5 ng/μL, and 1 μL was used in real-time PCR reactions to confirm the enrichment for hydroxymethylated region. The library was denatured with 0.1 M NaOH to generate single-stranded DNA molecules, and loaded onto channels of the flow cell (8 pM), and amplified in situ using TruSeq Rapid SR Cluster Kit (#GD-402-4001, Illumina). High-throughput sequencing was performed on the HiSeq 2000 (Illumina) using the TruSeq Rapid SBS Kit (#FC-402-4001, Illumina).

### Bioinformatic analysis of MBD-seq

Sequenced reads were mapped to the reference human genome (GRCh37, hg19) using Bowtie (v0.12.7) [49]. Significantly enriched regions/peaks of methylation were determined using model-based analysis of ChIP-seq (MACS) algorithm [50], by comparing bound, enriched samples to input, non-enriched samples. Annotation was performed using ChIPpeakAnno R package (v.1.12.0) [51] and a customized version of Annovar program [52] with RefSeq genes to determine specific genomic features: promoter regions (2.5 kb upstream and 500 bp downstream from the nearest TSS), genic (further defined as 500 bp downstream from the nearest TSS), exons, introns, and intergenic (defined as greater than 100 kb up/downstream from the nearest TSS). UCSC CpG island definitions were used to define CpG islands. RWPE-1 DNase-seq data from the Encyclopedia of DNA Elements (ENCODE) project [GEO accession: GSM1008595] was correlated to MACS peak sets in both RWPE-1 and 22Rv1 cells. The genomic feature, RWPE-1 UCSC DNAseI peaks, was defined as within 5 kb of a DNase peak. The following options for ChiPpeakAnno were used: PeakLocForDistance=“middle,” FeatureLocForDistance=“middle” for DNase I HS and CpG data, and FeatureLocForDistance=“TSS” for RefSeq gene annotations. MACS data was further analyzed using DiffBind R package (v.1.12.0) [53] to determine three consensus peak sets by requiring that peaks from one, two, or all three replicates be present at the same location. The third consensus peak set was subsequently defined as differentially methylated regions (DMRs) between RWPE-1 and 22Rv1 cells (fold change >1, FDR < 0.1).

### Bioinformatic analysis of hMeSeal-seq

Sequenced reads were mapped to the reference human genome (GRCh37, hg19) using Bowtie (v0.12.7) [49]. Repitools package [54] was used as an enrichment diagnostic screen of sequenced samples, enriched and input. Significantly enriched regions/peaks of hydroxymethylation were determined using model-based analysis of ChIP-seq (MACS) algorithm [50], by comparing bound, enriched samples to input, non-enriched samples. Annotation was performed using ChIPpeakAnno R package (v.1.12.0) [51] and a customized version of Annovar program [52] with RefSeq genes to determine specific genomic features: promoter, genic, exons, introns, and intergenic (previously described for MBD-seq). UCSC CpG island definitions were used to define CpG islands. RWPE-1 DNase-seq data from the ENCODE project [GEO accession: GSM1008595] was correlated to MACS peak sets in both RWPE-1 and 22Rv1 cells. The genomic feature, RWPE-1 UCSC DNAseI peaks, was defined as within 5 kb of a DNase peak. MACS data was further analyzed using DiffBind R package (v.1.12.0) [53] to determine two consensus peak sets by requiring one or both MACS peaks to be present for a consensus peak to be called. The first consensus peak set was subsequently defined as differentially hydroxymethylated regions (DHMRs) between RWPE-1 and 22Rv1 cells (fold change >1).

### Bioinformatic analysis of hMeDIP-seq

Sequencing image analyses and base calling were performed using Off-Line Basecaller software (OLB V1.8). After passing Solexa CHASTITY quality filter, sequenced reads were mapped to the reference human genome (GRCh37, hg19) using Bowtie (V2.1.0) [49]. Significantly enriched regions/peaks of hydroxymethylation were determined using model-based analysis of ChIP-seq (MACS, V2) algorithm [50], by comparing immunoprecipitated, enriched samples to input, non-enriched samples (*p* value < 10^−4^). Annotation was performed using the UCSC RefSeq database, where peaks were mapped to the nearest gene and specific genomic features were determined: promoter (2500 kb upstream and 500 bp downstream from the TSS); gene body (500 bp downstream of the TSS to the TTS), including introns and exons; and intergenic (remaining regions not defined as “promoter” or “gene body” located more than 100 kb from the nearest TSS). UCSC CpG island definitions were used to define CpG islands. RWPE-1 DNase-seq data from the ENCODE project [GEO accession: GSM1008595] was correlated to MACS peak sets in both RWPE-1 and 22Rv1 cells. The genomic feature, RWPE-1 UCSC DNAseI peaks, was defined as within 5 kb of a DNase peak.

### Statistical analysis: correlation between genome-wide DNA methylation and hydroxymethylation marks

Specific regions/peaks from genome-wide MBD-Seq and hMeSeal-seq data where DNA hydroxymethylation overlapped methylation, hydroxymethylation overlapped hydroxymethylation, and methylation overlapped hydroxymethylation for RWPE-1 and 22Rv1, respectively, were identified and stratified by genomic feature as described previously. Significant proportional difference for modified regions from the expected proportion of overall RWPE-1 and 22Rv1 marks, such that the frequency of occurrence of modified regions could be explained by neither random change in RWPE-1 marks or by random distribution of 22Rv1 marks, was assessed using chi-square test.

### Statistical analysis: correlation between genome-wide DNA methylation or hydroxymethylation and gene expression

Microarray RNA expression data were downloaded from GEO DataSets for RWPE-1 [GEO accession: GSM375783] and 22Rv1 [GEO accession: GSE36135]. Affymetrix probe identifications were linked to gene names using the R package (hgu133plus2.db, v.3.0.0), and a single expression value per gene was selected (a calculated average between replicates). The resulting dataset was sorted by expression and divided into three equal subsets/tiers of genes with low, medium, and high expression. Consensus peaks from MBD-seq (three sets) and hMeSeal-seq (two sets) analyses were intersected with each of the three tiers of genes, from microarray datasets. Peaks were obtained when found to overlap or flank genes with low (zero), moderate, or high expression. Each of the resulting datasets (15 sets) was further stratified into subsets of peaks differing in their location according to genomic features (previously defined for MBD-seq). In addition to the count of peaks for each of the peak sets defined above, the corresponding count of associated RefSeq coding genes was also recorded. The strength of association between peak or gene count and expression level was assessed using chi-square test, Pearson’s correlation coefficient, and Fisher’s exact two-tailed test, using R package (v.3.1.0). Fisher’s exact test was performed comparing tier 1 (low expression) to tier 3 (high expression) gene/peak sets.

### Pathway analysis of MBD-seq and hMeSeal-seq datasets correlated with gene expression

Genomic region lists were generated to represent significant differentially methylated or hydroxymethylated regions (DMRs or DHMRs), overlapping specific genomic features, identified by comparing RWPE-1 and 22Rv1 cells. DMR or DHMR lists were further stratified according to correlation with microarray RNA expression datasets (as described above). Pathway enrichment analysis was performed on the genomic region lists using the Genomic Regions Enrichment of Annotations Tool (GREAT) [55]. The background file submitted contained a complete list of total methylated or hydroxymethylated regions in both RWPE-1 and 22Rv1 datasets (no significance threshold applied). GREAT results were represented as an enrichment map (*p* < 0.05, FDR < 0.1, Jaccard’s similarity coefficient < 0.25) [56] generated in the visualization software, Cytoscape (v3.1.0) [57]. Groups of related pathways (nodes) were identified using the MCODE cluster app and were then manually summarized (circled and labeled in figures) [58]. Additionally, GREAT results were organized into bar graphs according to the recommended statistic of −log_10_ (raw hypergeometric *p* value) for ranking. Additionally, NGS (raw peaks) and microarray (raw gene expression) data were visualized using Circos software [59].

### Locus-specific DNA hydroxymethylation detection using hMeSeal-qPCR

Following hMeSeal technique performed on a separate biological replicate from hMeSeal-Seq (previously described), DNA hydroxymethylation levels were analyzed by RT-qPCR using the 7500 Real-Time PCR Instrument (Applied Biosystems). Hydroxymethylation levels were calculated using 2^−∆∆Ct^ method, relative to 2.7 % of input genomic DNA, and normalized to 0.027 % of input genomic DNA. A negative control reaction was performed according to the Hydroxymethyl Collector protocol (Active Motif), where UDP-azide-glucose was excluded from the glucosylation reaction. In addition, 2.5 μg of sheared genomic DNA was subjected to each glucosylation reaction, for both sample and negative control reactions. The PCR assays comprised of 5 μl of PerfeCTa® SYBR® Green FastMix®, Low ROX (Quanta Biosciences), 2 μl of bound DNA elution (hydroxymethylated or negative control), and 5 μM of each primer, with a total volume of 10 μl. The 2.7 and 0.027 % of input PCR assays were completed using the identical conditions, and input DNA stocks were prepared at 33.3 and 0.33 ng/μl, respectively. All PCR assays included a non-template control, using UltraPure Distilled Water (Invitrogen) as the template. The PCR conditions were as follows: 30 s at 95 °C, 40 cycles of denaturation for 5 s at 95 °C, and annealing for 30 s at 60 °C. Primers were designed using the specifications recommended by Hydroxymethyl Collector protocol (Active Motif) and targeted differentially hydroxymethylated regions (DHMRs) identified by both hMeSeal-seq and hMeDIP-seq. The sequences of primers amplifying: *RASGEF1a* are 5′-GCA TGT TCC TTG AAC TGT GA-3′ (forward), 5′-TCA CAC CCT TCC CAA CAC TA-3′(reverse); *HINT1* are 5′- CAT ATC CAA ATT GCC AGG AT-3′ (forward), 5′- GCT GAC TTT GCT TTC AGA CC-3′ (reverse); *CUL2* are 5′-GGG GTG CAA TAT CTC ACT GT-3′ (forward), 5′-GCT TGG AGA AGA CAC ACA AA-3′ (reverse); and *HOXD8* are 5′-AAC TTG CGG TCG TCT GCC CT-3′ (forward), 5′-ACA GAA ACG TTC TGA GGC GGG AAA-3′ (reverse). PCR reactions were performed across three technical replicates.

### Accession numbers

The data sets supporting the results of this article are available in the Gene Expression Omnibus (GEO) repository, under accession number GSE74464.

## Additional file

Additional file 1: Co-occurrence of methylation and hydroxymethylation in pathway regulation. This file lists all pathways found to be co-occurrent between 5mC- and 5hmC-enriched regions within each cell line, providing the genomic features and gene lists associated with enrichment for each mark.
