# Supplementary material for: Dynamic interplay between locus-specific DNA methylation and hydroxymethylation regulates distinct biological pathways in prostate carcinogenesis
Source: Clin Epigenetics. 2016 Mar 15;8:32. doi: 10.1186/s13148-016-0195-4 (PMC4791926; doi:10.1186/s13148-016-0195-4)
Supplement: Additional file 1: — Co-occurrence of methylation and hydroxymethylation in pathway regulation. This file lists all pathways found to be co-occurrent between 5mC- and 5hmC-enriched regions within each cell line, providing the genomic features and gene lists associated with enrichment for each mark. [file 13148_2016_195_MOESM1_ESM.zip › Supplementary Methods and Figures.docx]

# Supplemental Materials and Methods

## Global DNA Hydroxymethylation Detection using Dot Blot Assay

Human 5mC and 5hmC genomic DNA standards (Zymo) and mouse tissue-derived genomic DNA of known 5mC and 5hmC percentage (Zymo) were used to optimize the dot blotting protocol. DNA was denatured in 0.4 M NaOH/10 mM EDTA for 10 min at 95°C and spotted onto a positively charged nylon membrane (Roche) using a Dot Blot apparatus (Durocher lab, Mount Sinai Hospital), and cross-linked by alkaline fixation. Membrane was washed with 2X saline-sodium citrate (SSC) buffer, air dried for 1 hour, and blocked with 5% non-fat milk in tris-buffered saline and tween 20 (TBS-T) for 1 hour, at room temperature. Following, the membrane was incubated with anti-5hmC antibody (Active Motif), overnight at 4°C. After washing, membrane was incubated with horseradish peroxidase (HRP)-conjugated anti-rabbit IgG secondary antibody (Life Technologies) for 1 hour. Membrane was treated with enhanced chemiluminescence (ECL) for visualization. Relative quantification was performed using 0.04% methylene blue in 0.3 M sodium acetate, pH 5.2 and ImageJ software.

## Validation of MBD-seq Results using Methylation Microarrays

MBD-seq datasets and identified regions were compared to two independent methylation microarrays previously performed in our lab (Supp. Tables X-Y). Firstly, an Agilent Human CpG Island methylation microarray contained 22Rv1 cells was analyzed [1]. In this study, untreated 22Rv1 cells were compared to those treated with 5-aza-2’–deoxycytidine (DAC), a demethylating agent. The top 65 ranking regions/probes within the hypomethylated fraction were assessed for overlap with MBD-seq dataset of 22Rv1. Secondly, data from an Illumina Infinium HumanMethylation450 BeadChips methylation microarray that sampled both untreated RWPE-1 and 22Rv1 cells was analyzed [1]. Raw beta values were used to determine regions/probes with high methylation signal (0.9 to 1) [2, 3]. Regions assessed were not normalized or tested for statistical significance between RWPE-1 and 22Rv1 cells. Probes were ranked based on beta value and 12 probes were chosen for comparison with MBD-seq, based on the beta value observed for the other cell line.

**Supplementary Figure Legends**

**Supplementary Figure S1. Genome-wide methylation, hydroxymethylation, and expression profiling in normal prostate versus prostate cancer cell lines.** Circos representation of MBD-Seq, hMeSeal-seq, and publicly available microarray expression data for RWPE-1 (*right semi-circle*; normal prostate, labeled RW) and 22Rv1 (*left semi-circle;* prostate cancer, labeled RV) cells. Measured tickmarks indicate Mbp on each labelled human chromosome. Each chromosome (distinct horizontal bars) is labeled and represented by a different color. Tile tracks represent methylation peaks *(orange)*, expression data *(red, blue, and green)*, or hydroxymethylation peaks *(purple)* detected at each tile position along a given chromosome. Stacked tile height indicates relative enrichment at any given chromosomal position for methylation or hydroxymethylation tracks, or multiple genes at a given locus of the expression track. Tile color within the expression track indicates whether genes were identified as being within the lowest tier of expression *(red)*, the middle tier *(blue)*, or the highest *(green)*. Hydroxymethylation changes in cancer are depicted in the center of the diagram by lines connecting red (22Rv1) and blue (RWPE-1) horizontal bars representative of chromosomes. *Red lines*: hydroxymethylation “absence” regions where 5hmC marks were observed in RWPE-1 and 5mC marks observed in 22Rv1. *Yellow lines*: hydroxymethylation "retention", where 5hmC was detected in both cell lines. *Blue lines*: hydroxymethylation "gain", where 5mC was detected in RWPE-1 and 5hmC was detected in cancer.

**Supplementary Figure S2. Diagnostic enrichment plot of hMeSeal-seq samples from RWPE-1 and 22Rv1.** Enrichment diagnostic graphs comparing the curve of input (non-enriched) sample (green, blue lines) to biological replicate 2 (black, red lines) in RWPE-1 and 22Rv1 cells respectively.

**Supplementary Figure S3. Dot blot analysis of global levels of hydroxymethylation in RWPE-1 and 22Rv1 cells. (a)** Representative micrographs of dot blotting assays of RWPE-1 and 22Rv1 genomic DNA, where 500ng was serially diluted and incubated with anti-5hmC antibody. Methylene blue staining was used as total genomic DNA loading control. **(b)** Global detection and quantification of 5hmC marks at 500ng dilution, relative to 5hmC intensity in RWPE-1 cells are shown as ± standard deviation from 2 technical replicates.

**Supplementary Figure S4. Validation of specificity for HydroxyMethyl Collector kit controls.** hMeSeal-qPCR was performed on three technical replicates of RWPE-1 DNA spiked with 5hmC, 5mC, and C controls using the ΔΔC_t_ method with normalization to 10% input. Detection of hydroxymethylated, methylated, or unmodified cytosine oligomers in bound fractions as compared to unbound fractions is shown.

**Supplementary Figure S5. Comparison of called peak distribution between hMeSeal-Seq and hMeDIP-Seq in RWPE-1 cells.** Percentage distribution of absolute hydroxymethylated peaks called in single replicate by MACS between hydroxymethyl-selective sequencing performed on two different platforms in RWPE-1 cells. Asterisks indicate significant (p<0.05) differences between peak proportions as determined by chi-square test.

**Supplementary Figure S6. Validation of specificity for MethylMiner kit controls.** MBD-qPCR was performed on three technical replicates of control K-562 DNA spiked with 5hmC, 5mC, and C controls using the ΔΔC_t_ method with normalization to 10% input. Detection of hydroxymethylated, methylated, or unmodified cytosine oligomers in bound fractions and unbound fractions as compared to negative controls is shown.

**Supplementary Figure S7. Differential methylation and hydroxymethylation peak distribution between RWPE-1 and 22Rv1 cell lines. (a)** Percentage distribution of absolute methylation peaks called across three replicates by MACS following MBD-Seq in normal prostate and prostate cancer cell lines. **(b)** Percentage distribution of absolute hydroxymethylation peaks called in single replicate by MACS following hMeSeal-Seq in normal prostate and prostate cancer cell lines. Asterisks indicate significant (p<0.05) differences between peak proportions as determined by chi-square test.

**Supplementary Figure S8. Correlation of locus-specific hydroxymethylcytosine marks identified via hMeDIP-Seq with gene expression.** Regions displaying absolute hydroxymethylation peaks called by MACS in RWPE-1 as determined by hMeDIP-Seq stratified by genomic feature and relative abundance of hydroxymethylation marks across equal expression tiers derived from microarray analysis. Asterisks represent significant (p<0.05) correlation between the presence of 5hmC marks in a given genomic feature and gene expression as determined by chi-square 2 by k trend test.

**Supplementary Figure S9. Pathway regulation by methylation marks in normal prostate and prostate cancer.** Pathway enrichment annotations from GREAT for methylation within 22Rv1 cells, overlapping **(A)** CGI or **(B)** exonic regions; and RWPE-1 overlapping **(C)** exonic regions*.* Pathways further represented by genes within the highest (*High expression)* and lowest tier of expression (*Low expression*).

**Supplementary Figure S10. Pathway regulation by methylation marks in normal prostate and prostate cancer.** Pathway enrichment annotations from GREAT for methylation in intronic regions within **(A)** 22Rv1 or **(B)** RWPE-1 cells. Pathways further represented by genes within the highest (*High expression)* and lowest tier of expression (*Low expression*).

**Supplementary Figure S11. Pathway regulation by hydroxymethylation marks in normal prostate and prostate cancer.** Pathway enrichment annotations from GREAT for hydroxymethylation overlapping CGIs within **(A)** RWPE-1 or **(B)** 22Rv1 cells. Pathways further represented by genes within the highest (*High expression)* and lowest tier of expression (*Low expression*).

**Supplementary Figure S12. Pathway regulation by intergenic hydroxymethylation marks is not observed in normal prostate cells.** Pathway enrichment annotations from GREAT for hydroxymethylation within intergenic features within the lowest tier of expression within RWPE-1 cells.

**Supplementary Figure S13. Representative genes for validation are not methylated in either cell line.** MBD-qPCR was performed on three technical replicates using the ΔΔC_t_ method with normalization to 0.03% input.**(A)** Methylation in RWPE-1 for MethylMiner-enriched fractions versus negative control reaction without MBD-biotin, as compared to HOXD8 positive control. **(B)** Methylation in 22Rv1 as compared to negative control reactions without MBD-biotin. The HOXD8 locus tested is unmethylated in 22Rv1, and thus could not be used as a control.

**Supplementary Figure S14. HOXD8 promoter methylation identified by MBD-Seq in RWPE-1 cells.** IGV Genome Browser representation of absolute methylation peaks for the promoter region of HOXD8 amplified in hMeSeal-qPCR, called across three replicates from MACS following MBD-Seq in RWPE-1 and compared to unenriched input control. Peak strength indicated by height of representative peak within each replicate or input control, showing robust methylation enrichment in HOXD8 in two out of three replicates. *Bottom:* Representative diagram indicating the position of the hMeSeal-qPCR amplified segment depicted relative to the overall location of the gene (figure not to scale). Gene diagram adapted from UCSC Genome Browser.

**Supplementary Figure S1**

**
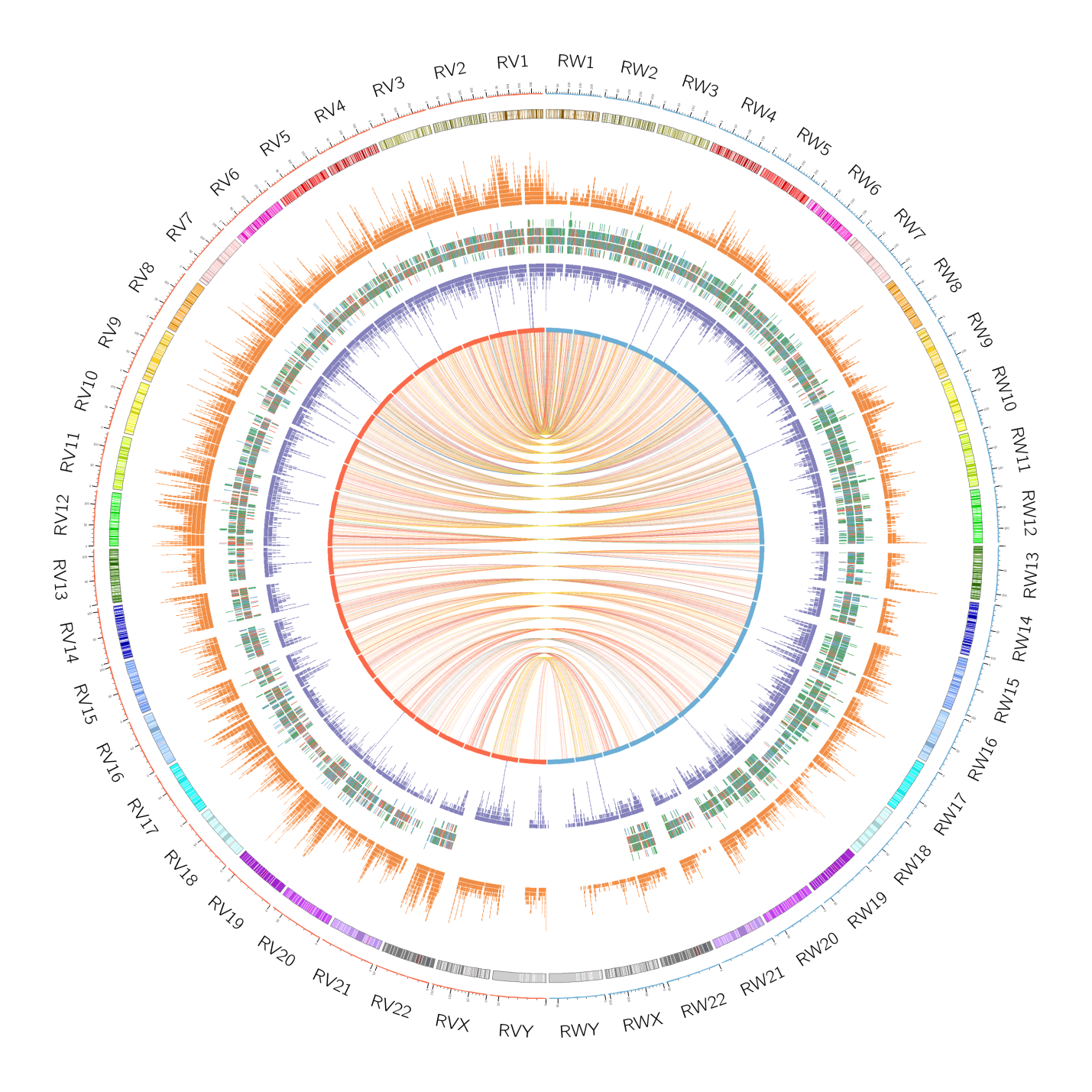
**

**Supplementary Figure S2**

**Supplementary Figure S3**

**
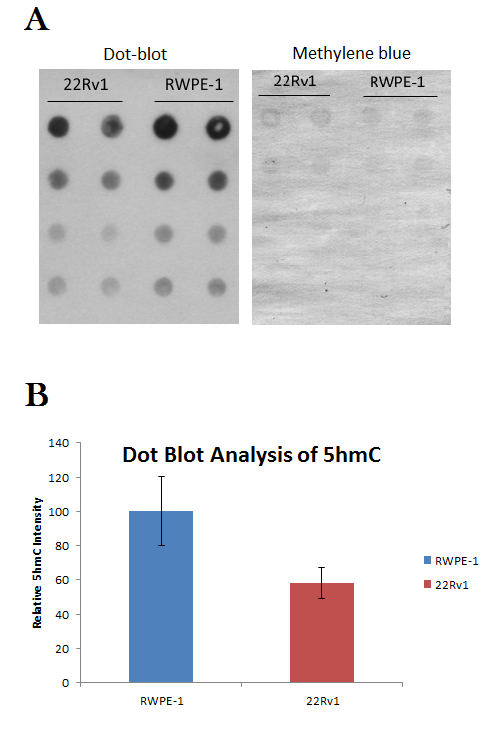
**

**Supplementary Figure S4**

**Supplementary Figure S5**

**Supplementary Figure S6**

**Supplementary Figure S7**

*

**Supplementary Figure S8**

**
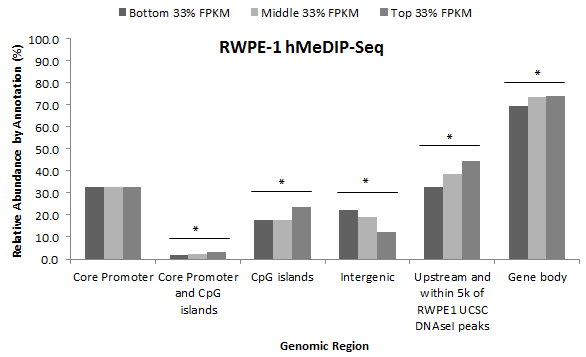
**

**Supplementary Figure S9**

**
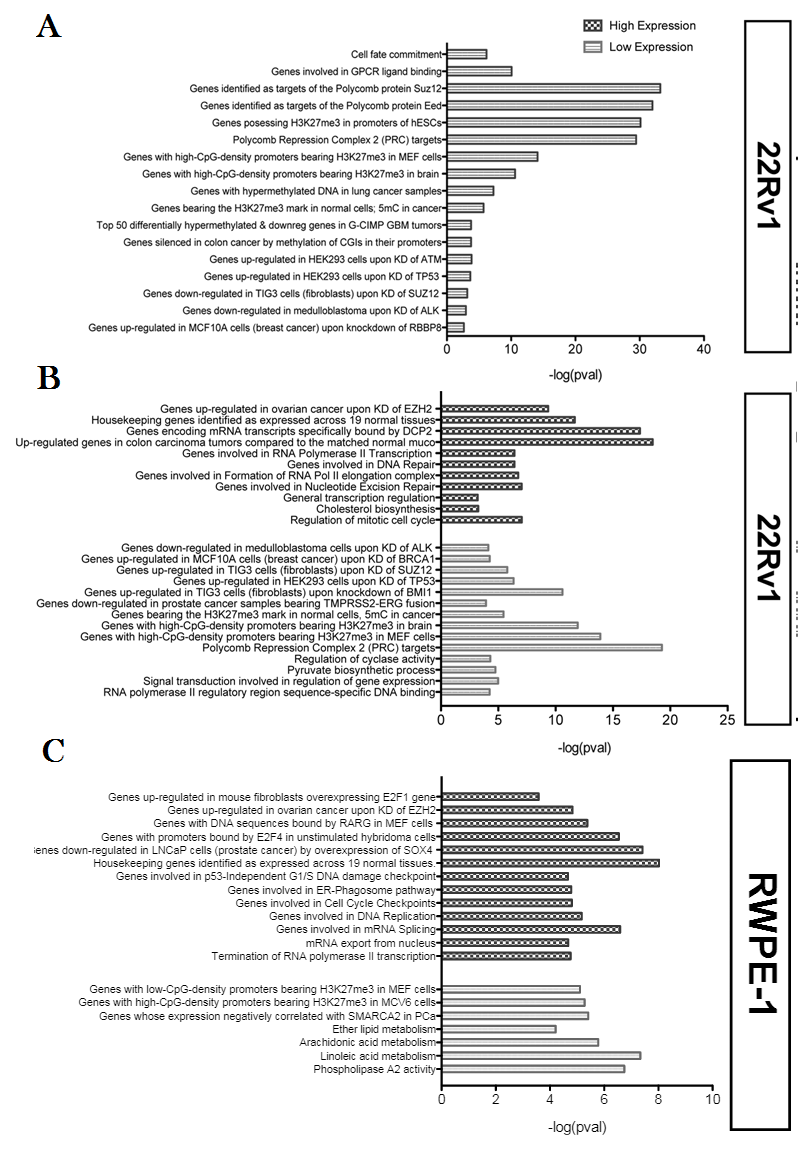
**

**Supplementary Figure S10**

**
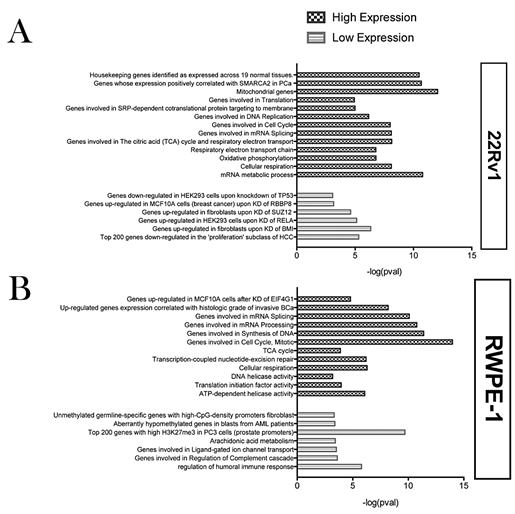
**

**Supplementary Figure S11**

**
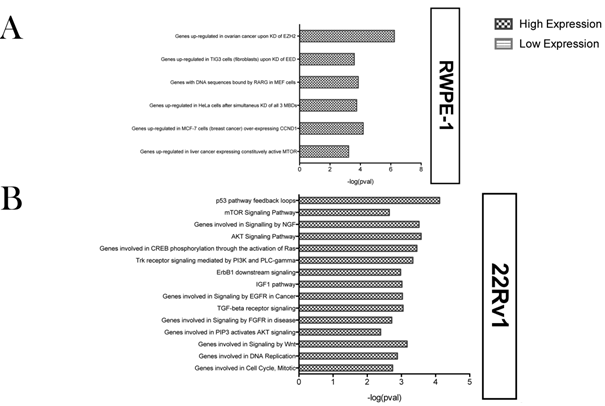
**

**Supplementary Figure S12**

**
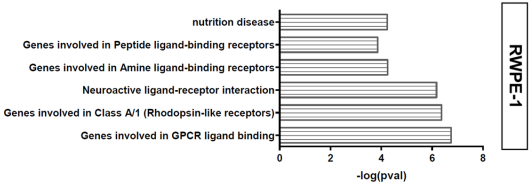
**

**Supplementary Figure 13**

**
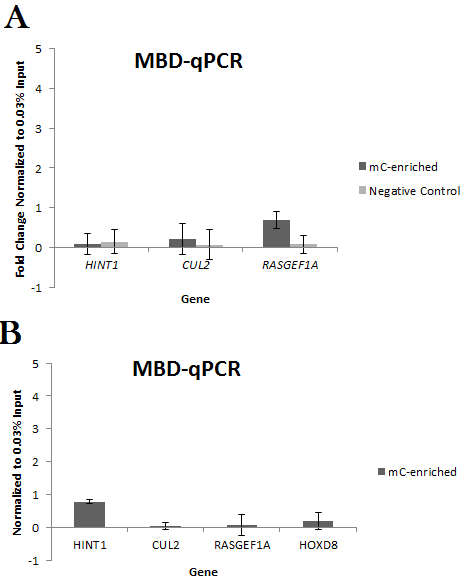
**

**Supplementary Figure S14**

**
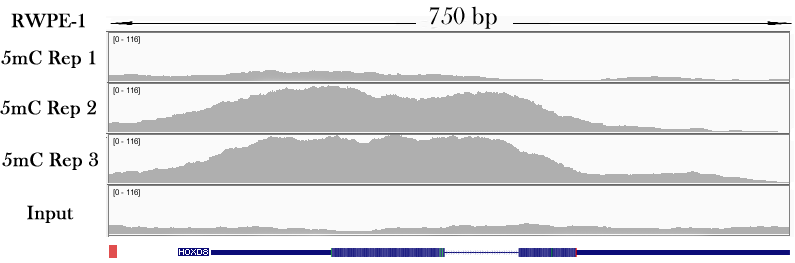
**

**Supplementary References**

1. White-Al Habeeb NM, Ho LT, Olkhov-Mitsel E, Kron K, Pethe V, Lehman M, et al. Integrated analysis of epigenomic and genomic changes by DNA methylation dependent mechanisms provides potential novel biomarkers for prostate cancer. Oncotarget. 2014; 5(17):7858-69.
2. Bibikova M, Lin Z, Zhou L, Chudin E, Garcia EW, Wu B, et al. High-throughput DNA methylation profiling using universal bead arrays. Genome Res. 2006; 16(3):383-93.
3. Bibikova M, Fan JB. GoldenGate assay for DNA methylation profiling. Methods Mol Biol. 2009; 507:149-63.
4. Sakharkar MK, Chow VT, Kangueane P. Distributions of exons and introns in the human genome. In Silico Biol. 2004; 4(4):387-93.
5. Han L, Zhao Z. CpG islands or CpG clusters: how to identify functional GC-rich regions in a genome? BMC Bioinformatics. 2009; doi: 10.1186/1471-2105-10-65.
6. Saxonov S, Berg P, Brutlag DL. A genome-wide analysis of CpG dinucleotides in the human genome distinguishes two distinct classes of promoters. Proc Natl Acad Sci. 2006; 103(5):1412-7.
7. Illingworth RS. Gruenewald-Schneider U, Webb S, Kerr AR, James KD, Turner DJ, et al. Orphan CpG islands identify numerous conserved promoters in the mammalian genome. PLoS Genet. 2010; doi: 10.1371/journal.pgen.1001134.
