# Supplementary material for: Dynamic interplay between locus-specific DNA methylation and hydroxymethylation regulates distinct biological pathways in prostate carcinogenesis
Source: Clin Epigenetics. 2016 Mar 15;8:32. doi: 10.1186/s13148-016-0195-4 (PMC4791926; doi:10.1186/s13148-016-0195-4)
Supplement: Additional file 1: — Co-occurrence of methylation and hydroxymethylation in pathway regulation. This file lists all pathways found to be co-occurrent between 5mC- and 5hmC-enriched regions within each cell line, providing the genomic features and gene lists associated with enrichment for each mark. [file 13148_2016_195_MOESM1_ESM.zip › Supplementary Tables.docx]

**Supplementary Table S1. Non-random distribution of methylation and hydroxymethylation peaks across the RWPE-1 and 22Rv1 genomes.**

|  |  |  |  | RWPE-1 | | 22Rv1 | | RWPE-1 | | 22Rv1 | |
| --- | --- | --- | --- | --- | --- | --- | --- | --- | --- | --- | --- |
|  |  |  |  | hMeSeal-Seq | | | | MBD-Seq | | | |
| Genomic Feature | Total Length (bp) | Total CpG Length (bp) | Percentage of Total (%) | Percentage of Total (%) | p-value | Percentage of Total (%) | p-value | Percentage of Total (%) | p-value | Percentage of Total (%) | p-value |
| Total | 3,017,700,646^4^ | 27,159,306^6^ |  |  |  |  |  |  |  |  |  |
| Exon | 39,841,315^4^ | 1,115,557^6^ | 4.107457 | 3.059509 | 0.585 | 1.923896 | 0.13 | 16.08554 | **0.001** | 15.17912 | **0.002** |
| Intron | 1,123,657,235^4^ | 11,236,572^6^ | 41.37283 | 33.13034 | 0.095 | 30.51446 | **0.023** | 35.87851 | 0.272 | 37.30704 | 0.445 |
| Promoter (est) | 79,692,000^4^ | 3,347,064^6^ | 12.32382 | 2.286357 | **<0.0001** | 1.223744 | **<0.0001** | 3.32617 | **<0.0001** | 4.552711 | **<0.0001** |
| Intergenic (est) | 1,854,202,096^4^ | 16,687,819^6^ | 61.4442 | 26.54266 | **<0.0001** | 31.87215 | **<0.0001** | 14.1989 | **<0.0001** | 12.98442 | **<0.0001** |
| CGI (est) | 41,184,000^5^ | 8,236,800^7^ | 30.32773 | 3.079367 | **<0.0001** | 1.789954 | **<0.0001** | 9.653866 | **<0.0001** | 9.812824 | **<0.0001** |

**Supplementary Table S2. Interrogation of internal controls to verify the specificity of the hMeDIP antibody.**

| **Sample** | Input DNA (Ct) | hMeDIP DNA (Ct) | **%(hMeDIP/Input)** | Negative Control (IgG) Ct | **%(Negative Control/Input)** | **Fold Enrichment** |
| --- | --- | --- | --- | --- | --- | --- |
| **RWPE-1-gDNA-p56-Sample1** | 23.721 | 23.876 | 17.963 | NA | 8.05E-03 | 2.23E+03 |
| **RWPE-1-gDNA-p56-Input** | 23.868 | 23.821 | 20.662 | NA | 8.91E-03 | 2.32E+03 |

**Supplementary Table S3. Correlation of methylation and hydroxymethylation marks with expression in RWPE-1 cells.**

|  | Methylation (MBD-Seq) | | | | Hydroxymethylation (hMeSeal-Seq) | | | |
| --- | --- | --- | --- | --- | --- | --- | --- | --- |
|  | Relative gene counts | | | RWPE-1 | Relative gene counts | | | RWPE-1 |
|  | bottom 33% FPKM | median 33% FPKM | top 33% FPKM | Chi^2^ P-value | bottom 33% FPKM | median 33% FPKM | top 33% FPKM | Chi^2^ P-value |
| Core Promoter | 16.6 | 16.7 | 17.9 | 0.17314 | 10.2 | 10.5 | 12.4 | **0.0027** |
| Core Promoter CGIs | 6.6 | 5.5 | 3.1 | **4.23E-15** | 3.5 | 4.8 | 6.0 | **2.36E-06** |
| CpG islands | 33.8 | 30.7 | 26.9 | **3.61E-12** | 10.2 | 12.0 | 13.9 | **4.50E-06** |
| Upstream | 42.4 | 43.4 | 44.0 | 0.30669 | 42.9 | 41.0 | 39.3 | **0.00524** |
| Upstream and within 5k of RWPE1 USCS DNAseI peaks | 23.2 | 26.9 | 31.9 | **5.93E-20** | 20.4 | 21.6 | 25.5 | **6.17E-08** |
| Gene body | 69.4 | 81.6 | 84.2 | **6.64E-72** | 58.4 | 68.0 | 66.9 | **7.37E-20** |

**Supplementary Table S4. Correlation of methylation and hydroxymethylation marks with expression in 22Rv1 cells.**

|  | Methylation (MBD-Seq) | | | | Hydroxymethylation (hMeSeal-Seq) | | | |
| --- | --- | --- | --- | --- | --- | --- | --- | --- |
|  | Relative gene counts | | | 22Rv1 | Relative gene counts | | | 22Rv1 |
|  | bottom 33% FPKM | median 33% FPKM | top 33% FPKM | Chi^2^ P-value | bottom 33% FPKM | median 33% FPKM | top 33% FPKM | Chi^2^ P-value |
| Core Promoter | 35.7 | 32.6 | 30.4 | **< 0.0001** | 3.1 | 2.8 | 2.8 | 0.8455 |
| Core Promoter CGIs | 20.2 | 13.0 | 8.3 | **< 0.0001** | 0.4 | 0.9 | 1.1 | 9.72E-02 |
| CpG islands | 50.9 | 46.0 | 29.4 | **< 0.0001** | 3.3 | 3.4 | 3.6 | 8.72E-01 |
| Intergenic | 16.4 | 11.0 | 11.0 | **< 0.0001** | 28.0 | 18.4 | 20.0 | **< 0.0001** |
| Upstream and within 5k of RWPE1 USCS DNAseI peaks | 56.3 | 72.3 | 70.2 | **< 0.0001** | 23.7 | 27.5 | 23.5 | **2.78E-02** |
| Gene body | 77.2 | 93.3 | 87.7 | **< 0.0001** | 45.7 | 57.6 | 48.3 | **< 0.0001** |

**Supplementary Table S5. Correlation of hydroxymethylation marks from hMeDIP-Seq with expression in RWPE-1 cells.**

|  | Hydroxymethylation (hMeDIP-Seq) | | | |
| --- | --- | --- | --- | --- |
|  | Relative gene counts | | | RWPE-1 |
|  | bottom 33% FPKM | median 33% FPKM | top 33% FPKM | Chi^2^ P-value |
| Core Promoter | 32.8 | 32.5 | 32.6 | 0.8868 |
| Core Promoter CGIs | 1.8 | 2.3 | 3.4 | **0.000288** |
| CpG islands | 17.6 | 17.6 | 23.4 | **1.92E-09** |
| Intergenic | 22.1 | 19.2 | 12.1 | **<2.2E-16** |
| RWPE1 UCSC DNAseI peaks | 32.8 | 38.6 | 44.8 | **<2.2E-16** |
| Gene body | 69.3 | 73.8 | 74.2 | **0.000681** |

**Supplementary Table S6. Locus-specific loss, gain, and retention of 5hmC in cancer.**

**Supplementary Table S7. Patterns of hydroxymethylation change between genomic features.**

|  | Intergenic | Genic | Exonic | Intronic | Promoter | CGI | DNAse |
| --- | --- | --- | --- | --- | --- | --- | --- |
|  | *5hmC "Loss" versus "Retention"* | | | | | | |
| 5hmC "Loss" | 8.8785047 | 45.56 | 19.86 | 23.3645 | 18.69159 | 42 | 51.869 |
| 5hmC "Retention" | 20.1923077 | 31.410256 | 0.64102564 | 30.449 | 2.5641 | 5.128205 | 1.6 |
| Chi-square test | **p=0.005** | **p=0.002** | **p<0.0001** | p=0.105 | **p<0.0001** | **p<0.0001** | **p<0.0001** |
|  | *5hmC "Loss" versus "Gain"* | | | | | | |
| 5hmC "Loss" | 8.8785047 | 45.56 | 19.86 | 23.3645 | 18.69159 | 42 | 51.869 |
| 5hmC "Gain" | 17.96875 | 34.375 | 3.906 | 29.688 | 2.34375 | 12.5 | 6.3 |
| Chi-square test | **p=0.019** | **p=0.014** | **p<0.0001** | p=0.143 | **p<0.0001** | **p<0.0001** | **p<0.0001** |
|  | *5hmC "Gain" versus "Retention"* | | | | | | |
| 5hmC "Gain" | 17.96875 | 34.375 | 3.906 | 29.688 | 2.34375 | 12.5 | 6.3 |
| 5hmC "Retention" | 20.1923077 | 31.410256 | 0.64102564 | 30.449 | 2.5641 | 5.128205 | 1.6 |
| Chi-square test | p=0.585 | p=0.577 | **p<0.0001** | p=0.922 | p=0.723 | **p<0.0001** | **p<0.0001** |

**Supplementary Table S8. Candidate genes chosen for validation.**
